# Incidental Finding of Attenuated Familial Adenomatous Polyposis

**DOI:** 10.7759/cureus.18237

**Published:** 2021-09-24

**Authors:** Siddharth Bhesania, Nikhila Chelikam, Navim Mobin, Sahar Ilyas, Neil Nimkar

**Affiliations:** 1 Internal Medicine, Overlook Medical Center, Summit, USA; 2 Internal Medicine, NewYork-Presbyterian Brooklyn Methodist Hospital, Brooklyn, USA; 3 Clinical Research, Icahn School of Medicine at Mount Sinai, New York City, USA; 4 Gastroenterology, NewYork-Presbyterian Brooklyn Methodist Hospital, Brooklyn, USA; 5 Hematology and Medical Oncology, Overlook Medical Center, Summit, USA; 6 Hematology and Medical Oncology, NewYork-Presbyterian Brooklyn Methodist Hospital, Brooklyn, USA

**Keywords:** gi polyps, communication and following up, apc gene mutation, familial adenomatous polyposis, attenuated adenomatous polyposis coli

## Abstract

Attenuated familial adenomatous polyposis (AFAP) or attenuated adenomatous polyposis coli (AAPC) is defined as the milder polyposis phenotype of classic familial adenomatous polyposis (FAP). FAP syndromes are caused by germline mutations in the adenomatous polyposis coli (APC) gene. AFAP is an inherited autosomal dominant with predominant mutations at the far proximal (5′) end of the APC gene. Unlike FAP, AFAP is characterized by the occurrence of fewer than 100 adenomas that are found mostly in the proximal part of the colon with a delayed progression to colorectal cancer (CRC). The lower risk of development of colorectal cancer and extra-intestinal neoplasms is likely attributable to under-diagnosis. However, 2-5% of all CRCs happen because of inherited syndromes which include both hereditary polyposis syndromes and hereditary nonpolyposis colorectal cancer syndrome (HNPCC) or otherwise known as Lynch syndrome (LS). Here, we present a case of a 64-year-old Polish-speaking female in whom an incidental finding of polyposis turned out to be a malignancy. Our patient had a positive family history of colon cancer. The delay in performing an annual colonoscopy with endoscopic polypectomy per AFAP surveillance guidelines was supposedly delayed due to lack of insurance and language barrier. There were no metastases and she was negative for the APC gene mutation. Pathology was significant for moderately differentiated adenocarcinoma with intact mismatch repair protein (MMRP) (expression of *MLH1, PMS2, MSH2*, and *MSH6*) genes immune-histochemical staining. The patient underwent a subtotal colectomy with ileostomy without any complications. This study aimed to emphasize that communication with the patient in their primary language is essential to gather all the data which can lead to an accurate diagnosis and to provide adequate care. Physicians are required to have a professional interpreter to acquire the appropriate medical history and be more vigilant in following up with the patient in order to provide comprehensive care.

## Introduction

Mutations in the adenomatous polyposis coli (APC) gene lead to the development of hereditary polyposis syndromes. Familial adenomatous polyposis (FAP), attenuated FAP (AFAP), and MUTYH-associated polyposis (MAP) are the three hereditary polyposis syndromes that have an increased potential for developing colorectal cancer. FAP syndrome mostly develops due to germline mutations of the APC gene on chromosome 5q21 and less frequently due to mutation in the MUTYH gene [1-3]. 

FAP due to APC gene mutation is inherited as an autosomal-dominant fashion and is classified based on the presence of greater than 100 colorectal adenomatous polyps and extra-intestinal manifestations [1-5]. The incidence of FAP is 1 in 8000 among the general population [6]. The prevalence was estimated to vary between 1 in 6850 and 1 in 31,250 live births (2.29 to 3.2 cases per 100,000 individuals) from the early 19th century to the early 20th century [1,7]. FAP appears to have various phenotypes and up to one-third of these newly diagnosed phenotypes do not fit into any of the previously identified FAP families. AFAP is one of the distinguishable phenotypes of FAP [1-3]. Unlike FAP, AFAP manifests by fewer than 100 colonic polyps with delayed development of adenomas and cancer as much as 10-20 years compared with typical FAP [1,3-5]. 

Without the prophylactic colectomy, the risk of colorectal cancer is almost 100% in cases of classical FAP by the age of 40 years [1-3], in comparison to 80% by the age of 80 years in cases of AFAP [8]. Through appropriate screening and surveillance, the morbidity and mortality of CRC can be improved [9]. The first aim of this study is to highlight the differences in FAP and AFAP presentation and approaches to therapy. The second aim is to emphasize the necessity of appropriate collection of medical history particularly when there is a language barrier and following up with the patient in order to provide comprehensive care.

## Case presentation

A 64-year-old Polish-speaking female scheduled for her regular dialysis session was found to have low hemoglobin of 6.1 g/dl in outpatient labs. She was immediately sent to the emergency department where she admitted to having increased fatigue as well as mild abdominal pain for three months. She also admitted that she was recently diagnosed with *Clostridium difficile* (*C. diff*) three months ago at another facility and was treated with vancomycin 125 mg PO with the resolvement of symptoms. She also reports experiencing a small amount of bright red blood with bowel movements which she attributed to external hemorrhoids. She denied any fevers, chills, nausea, headache, light-headedness, syncope, or other constitutional symptoms. Her past medical history included atrial fibrillation on Eliquis, type 2 diabetes mellitus, hypertension, hyperlipidemia, gout, glaucoma, coronary artery disease status post stent placement, recurrent *C. diff* colitis infection, and external hemorrhoids. The patient was undergoing dialysis for end-stage renal disease. Family history was significant for colon cancer in her mother. The patient never had any cancer screening before. She was reluctant to give any further medical and family history as she was an undocumented immigrant. 

Examination and investigations

On examination, the patient was hemodynamically stable with negative orthostatic blood pressure. Physical examination was remarkable for pallor conjunctiva as well as non-bleeding external hemorrhoids. The rectal examination was significant for maroon-colored guaiac positive stool. Given that she was found to have low hemoglobin of 6.1 mg/dl, high reticulocyte count as well as ferritin, the patient was given two units of the packed red blood cells (pRBC). Due to the extent of chronicity of symptoms, multiple comorbidities, and the requirement of dialysis, the patient was admitted for further care. She continued to receive all previous home medications except for aspirin and Eliquis. Dialysis sessions occurred as per schedule. The patient underwent EGD and colonoscopy given her anemia and maroon-colored stools. 

Outcome, intervention, and follow-up

**Figure 1:**
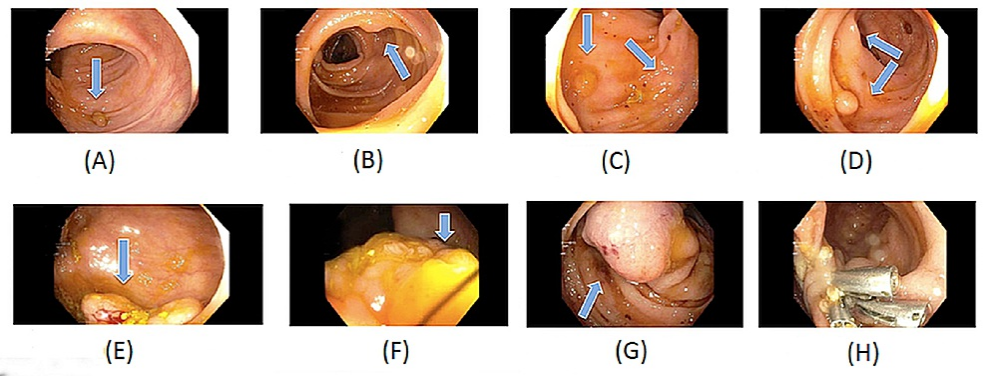
Colonoscopy showing polyposis with large polypoid lesions in the cecum, the proximal ascending colon, and the sigmoid colon. (A) Polyp at hepatic flexure, (B and C) multiple polyps in the transverse colon, (D) multiple polyps at splenic flexure, (E) 30 mm polypoid lesions in the cecum, (F) 30 mm polypoid lesions in the proximal ascending colon, (G) 40 mm polypoid lesion in the sigmoid colon, and (H) sigmoid polypectomy with hot snare and hemostasis clips

The patient was found to have multiple polyps in the entire colon with significant 30 mm polypoid lesions in the cecum and the proximal ascending colon and 40 mm lesion in the sigmoid colon (Figures 1A-1H). Polypectomy with hot snare and hemostasis clips was performed for the semi-pedunculated polyp in the sigmoid colon. Pathology was significant for moderately differentiated adenocarcinoma with intact MMRP immune-histochemical staining. EGD showed some gastritis and duodenal erosion but no signs of polyps or ulcers. No signs of metastasis on CT scan. Given the extent of the polyps, the patient underwent genetic testing which turned out to be negative for APC gene mutation. The patient was diagnosed with an attenuated variant of FAP as she was >50 years of age, has a positive family history of colon cancer in her mother, had <100 polyps and the presence of adenocarcinoma with intact MMRP gene. Due to the location and extent of the polyps and the presence of adenocarcinoma without any metastasis, she underwent a subtotal colectomy with ileostomy without any complications. 

After recovery, she was subsequently discharged home. She has had outpatient follow-up visits and is currently doing well. The patient is closely monitored by the surgery, gastroenterology, and hematology-oncology specialties on an outpatient basis.

## Discussion

To our knowledge, this is a unique case of an infrequent variety of AFAP that has not been reported yet. The initial step to approach AFAP is a clinical diagnosis which in turn helps in further management of the disease. In order to get an estimate of the risk of an inherited predisposition to cancer in patients with intestinal polyposis, obtaining a proper family history of cancer and premalignant lesions of the gastrointestinal tract is of utmost importance. Special focus on vertical transmission of the disease (from a generation to the next), age at diagnosis of cancers in the family (especially for first- and second-degree relatives) is also required. Of note, the histology of polyps removed in the large bowel can help in the management of AFAP [4]. 

AFAP is inherited in an autosomal dominant manner and arises from APC mutations from either the far proximal (5′) end of the gene, the far distal (3′) end of the gene, or in particular locations of exon 9. In fact, deletions of whole or partial genes would also give an attenuated phenotype. By the age of 80 years, the estimated cumulative risk of developing colorectal cancer in AFAP syndrome patients is 69% and the average age at which cancer is diagnosed is 58 years (range, 29-81 years) [1].

AFAP phenotypes were first described in the 1990s, however, the actual term was not used. Later on, AFAP was revised and recognized as a milder phenotypic variant of FAP [10]. Knudsen et al. proposed the following diagnostic criteria to help with the identification of AFAP syndromes: (1) a dominant mode of inheritance and (2) manifestation of less than 100 colorectal adenomas after the age of 25 years or (3) development of CRC after the age of 50 years; and (4) if a germline APC mutation is not detected, hereditary nonpolyposis colorectal cancer syndrome (HNPCC) or Lynch syndrome (LS) due to mutation in the mismatch repair protein (MMRP) genes should be ruled out by immunohistochemistry (IHC) staining [11].

The incidence of sporadic tumors in Lynch syndrome may occur more commonly in patients with MSH6 and PMS2 germline mutations than in patients with MLH1 or MSH2 mutations [9,12]. Our patient's IHC staining of DNA mismatch repair protein (MMRP) showed an intact nuclear expression of MLH1, PMS2, MSH2, and MSH6, however, she had a classic presentation of <100 polyps (approximately 15 sessile polyps) making this a case unique. 

FAP syndrome is often accompanied by extracolonic intestinal neoplasms that include gastric fundic gland hyperplastic polyps and duodenal adenomas [1,13]. The extraintestinal manifestations include but are not restricted to dental abnormalities, desmoid tumors, osteomas, congenital hypertrophy of the retinal pigmented epithelium, adrenal adenoma and cancers related to thyroid, liver, pancreas, and small intestine [1,7].

Progression of benign adenomas to colorectal cancer (CRC) in classic FAP is unavoidable if the colon is not prophylactically removed [1-5]. It is known that 2-5 % of all CRC occur due to inherited syndromes which include both hereditary polyposis syndromes and hereditary nonpolyposis colorectal cancer syndrome (HNPCC) or otherwise known as Lynch syndrome (LS) [14,15]. It is important to note that CRC is the third most common cause of cancer death in both men and women in the United States [9].

As opposed to classic FAP, colectomy is not always mandatory in asymptomatic patients of AFAP. It can be managed with an annual colonoscopy for surveillance of colorectal polyps and cancer. While the manifestation of upper GI polyps (gastric and duodenal) appear to be rare and develop at a later age compared to FAP, the risk of developing cancer is similar. Hence, endoscopic surveillance and polypectomy should also be performed at age 25-30 years and continued similarly to classical FAP [1-3].

Our patient, 64 years of age at the time of this admission, never had a colonoscopy. We suspected this scenario likely occurred due to a language barrier hindering detailed history (as her first language is Polish) as well as due to a lack of insurance and her undocumented status. Many studies have linked poor healthcare outcomes in patients who speak a foreign language. One study showed that most often physicians are relying on a bilingual healthcare worker and/or a family member for translation during the physical examination and/or history-taking process. Professional interpreters were used only about 5-39% of the time depending on the language [16].

## Conclusions

Attenuated FAP is characterized by multiple colonic polyps (average of 30), more proximally located polyps as well as a diagnosis of colon cancer at a later age. Early surveillance colonoscopy and EGD are good screening and monitoring tools however genetic testing is definitive. Management options depend on the severity of the symptoms and disease progression. Prophylactic colectomy is indicated for more than a hundred polyps, severely dysplastic polyps, or for multiple polyps larger than 1 cm. Our article reiterates some of the major issues in medicine, the importance of proper communication and following-up. Physicians must ensure that they are establishing appropriate communication by using professional interpreters when required. This reduces the probability of losing important healthcare plans due to improper language translation. Physicians also need to be keen on scheduling and following up on colonoscopies for their patients earlier than the normal population when there is an extensive family history of colon cancer, irrespective of the genetic mutation.

## References

[1] Syngal S, Brand RE, Church JM, Giardiello FM, Hampel HL, Burt RW (2015). ACG clinical guideline: genetic testing and management of hereditary gastrointestinal cancer syndromes. Am J Gastroenterol.

[2] Sokic-Milutinovic A (2019). Appropriate management of attenuated familial adenomatous polyposis: report of a case and review of the literature. Dig Dis.

[3] Plawski A, Nowakowska D, Podralska M, Lipinski D, Steffen J, Slomski R (2007). The AAPC case, with an early onset of colorectal cancer. Int J Colorectal Dis.

[4] Roncucci L, Pedroni M, Mariani F (2017). Attenuated adenomatous polyposis of the large bowel: present and future. World J Gastroenterol.

[5] Sant V, Reich E, Khanna L, Cao W, Kornacki S, Grucela A (2019). Attenuated familial adenomatous polyposis (AFAP) in a patient associated with a novel mutation in APC. BMJ Case Rep.

[6] Fearnhead NS, Britton MP, Bodmer WF (2001). The ABC of APC. Hum Mol Genet.

[7] Groen EJ, Roos A, Muntinghe FL (2008). Extra-intestinal manifestations of familial adenomatous polyposis. Ann Surg Oncol.

[8] Vasen HF, Möslein G, Alonso A (2008). Guidelines for the clinical management of familial adenomatous polyposis (FAP). Gut.

[9] Siegel RL, Miller KD, Sauer AG (2020). Colorectal cancer statistics, 2020. CA Cancer J Clin.

[10] Lynch HT, Smyrk TC (1998). Classification of familial adenomatous polyposis: a diagnostic nightmare. Am J Hum Genet.

[11] Knudsen AL, Bisgaard ML, Bülow S (2003). Attenuated familial adenomatous polyposis (AFAP). A review of the literature. Fam Cancer.

[12] Roth RM, Haraldsdottir S, Hampel H, Arnold CA, Frankel WL (2016). Discordant mismatch repair protein immunoreactivity in lynch syndrome-associated neoplasms: a recommendation for screening synchronous/metachronous neoplasms. Am J Clin Pathol.

[13] Spigelman AD, Williams CB, Talbot IC, Domizio P, Phillips RK (1989). Upper gastrointestinal cancer in patients with familial adenomatous polyposis. Lancet.

[14] Ma H, Brosens LA, Offerhaus GJ, Giardiello FM, de Leng WW, Montgomery EA (2018). Pathology and genetics of hereditary colorectal cancer. Pathology.

[15] Jasperson KW, Tuohy TM, Neklason DW, Burt RW (2010). Hereditary and familial colon cancer. Gastroenterology.

[16] Bischoff A, Hudelson P (2010). Communicating with foreign language-speaking patients: is access to professional interpreters enough?. J Travel Med.

